# Hippocampus and Insula Are Targets in Epileptic Patients With Glutamic Acid Decarboxylase Antibodies

**DOI:** 10.3389/fneur.2018.01143

**Published:** 2019-01-09

**Authors:** Mercè Falip, Laura Rodriguez-Bel, Sara Castañer, Jacint Sala-Padró, Júlia Miro, Sónia Jaraba, Carlos Casasnovas, Francisco Morandeira, Javier Berdejo, Mar Carreño

**Affiliations:** ^1^Epilepsy Unit, Department of Neurology, Hospital Universitari de Bellvitge, Hospitalet de Llobregat, Barcelona, Spain; ^2^PET Division, Institute of Diagnostic Imaging (IDI), Hospital de Bellvitge, Hospitalet de Llobregat, Barcelona, Spain; ^3^MRI Division, Institute of Diagnostic Imaging (IDI), Hospital de Bellvitge, Hospitalet de Llobregat, Barcelona, Spain; ^4^Neuromuscular Unit, Department of Neurology, Hospital Universitari de Bellvitge, Hospitalet de Llobregat, Barcelona, Spain; ^5^Immunology Unit, Biochemistry Department, Hospital Universitari de Bellvitge, Hospitalet de Llobregat, Barcelona, Spain; ^6^Department of Cardiology, Hospital Universitari de Bellvitge, Hospitalet de Llobregat, Barcelona, Spain; ^7^Epilepsy Unit, Department of Neurology, Hospital Clinic i Provincial, Barcelona, Spain

**Keywords:** temporal lobe epilepsy, glutamic acid decarboxylase antibodies, autoimmune epilepsy, limbic system, insula, hippocampus

## Abstract

**Background:** Antibodies to glutamic acid decarboxylase (GAD ab) have been found in patients with limbic encephalitis (LE) and chronic pharmacoresistant focal epilepsy (FE). The objectives of the study were to: (1) analyze the clinical and neuroimaging course of patients with FE+GAD ab, (2) compare these characteristics with a control group, and (3) describe the most affected cerebral areas with structural and functional imaging.

**Methods:** Patients with FE + high titers of GAD ab and a follow-up of at least 5 years were selected. Titers of serum GAD ab exceeding 2,000 UI/ml were considered high. Evolutive clinical and radiological characteristics were studied in comparison to two different control groups: patients with bilateral or with unilateral mesial temporal sclerosis (BMTS or UMTS) of a non-autoimmune origin.

**Results:** A group of 13 patients and 17 controls were included (8 BMTS, 9 UMTS). The most frequent focal aware seizures (FAS) reported by patients were psychic (5/13: 33%). Somatosensorial, motor, and visual FAS (4/13:32%) (*p*: 0.045), musicogenic reflex seizures (MRS), and a previous history of cardiac syncope were reported only patients (2/13:16% each) (*p*: NS). Comparing EEG characteristics between patients and controls, a more widespread distribution of interictal epileptiform discharges (IED) was observed in FE+ GAD ab patients than in controls (*p*:0.01). Rhythmic delta activity was observed in all controls in anterior temporal lobes while in patients this was less frequent (*p*: 0.001). No IED, even in 24 h cVEEG, was seen in 6 patients (46%).First MRI was normal in 4/5 (75%) patients. During the follow-up mesial temporal lobe (MTsL) sclerosis was observed in 5/8 (62%) of patients. All patients had abnormal FDG-PET study. MTL hypometabolism was observed in 10/11 (91%) patients, being bilateral in 7/11 (63%). In controls, this was observed in 16/17 (94%), and it was bilateral in 8/17 (47%) (*p*: NS). Insular hypometabolism was observed in 5/11 (45%) patients (*P*:0.002).

**Conclusions:** Clinical, EEG, and FDG-PET findings in FE+GAD ab suggest a widespread disease not restricted to the temporal lobe. Progressive MTL sclerosis may be observed during follow-up. In comparison to what is found in patients with non-autoimmune MTL epilepsy, insular hypometabolism is observed only in patients with GAD ab, so it may be an important diagnostic clue.

## Introduction

Gamma-aminobutyric acid (GABA) is the main inhibitor neurotransmitter in the mature brain. GABAergic interneurons represent 10–20% of neurons in the cortex and they play a critical role in modulating the output of the critical excitatory pyramidal neurons. Several groups of non-pyramidal GABAergic cells have been identified. Parvalbumin (PV)-expressing, fast-spiking basket cells form the most numerous class of cortical interneurons, whose perisomatic, basket-shaped axonal boutons exert exquisite control over the spiking activity of connected neurons (1).

Antibodies against GAD, the rate-limiting enzyme for the synthesis of GABA, were initially recognized in the serum and cerebrospinal fluid (CSF) of patients with stiff person syndrome (SPS), a rare central nervous system (CNS) disorder which produces rigidity, and cramps frequently associated with other autoimmune diseases, mainly type 1 diabetes mellitus (T1DM). Moreover, GAD ab is identified in about 80% of newly diagnosed T1DM patients, although at low titers compared with those found in SPS. Since then, high levels of GAD ab have also been described in several neurological disorders (2). Among patients with epilepsy, GAD ab has been found not only in patients with limbic encephalitis (LE) but also in patients with chronic temporal lobe epilepsy (TLE) (3). A case report has suggested that GAD ab can also produce epileptogenic areas outside the temporal lobe, in particular in the inferior rolandic area (4). In patients with acute debut presenting as LE, brain MRI usually shows signal and volume changes, bilaterally in most cases, in the temporolimbic structures. Chronic cases referred for epilepsy surgery are frequently rejected because of bilateral independent seizure onset zones in both temporal lobes (5).

One study using quantitative FLAIR analysis has suggested that the amygdala is more affected than the hippocampus in patients suffering LE of different etiologies including those related to GAD ab (6). Interestingly, in patients with LE due to GAD ab, MRI with voxel based morphometry performed 2 years after the onset of epilepsy showed no hippocampal volume reduction (7). A case report has also observed no hippocampal volume loss even 7 years after epilepsy debut (8).

In general there is little information about the long-term clinical and radiological outcome of patients with epilepsy +GAD ab. In addition, information about the cerebral areas which are more involved is also scarce, and it is not known whether the condition affects temporolimbic areas only or more widespread regions.

The aims of our study were to: (1) analyze both clinical and neuroimaging (structural and functional) characteristics of patients with focal epilepsy (FE) + GAD ab at epilepsy onset and during follow-up, (2) compare these characteristics with those of a control group of clinically similar patients without antibodies, and (3) describe the most affected cerebral areas in structural and functional imaging.

## Material and methods

### Patients and Controls

All patients with FE and high titers of GAD ab diagnosed at the Hospital Universitari de Bellvitge from May, 2006, to December, 2015, were selected. Only patients with a disease duration (from epilepsy onset) of more than 5 years were included in the study, in order to provide an adequate overview of the evolution of the disease.

In order to evaluate specific cerebral areas affected by GAD ab, FDG-PET studies performed in patients were compared with those from two different control groups: (1) a group of patients with unilateral mesial temporal lobe sclerosis (MTS) considered good surgical candidates after a congruent study with scalp video-EEG, surgically treated and seizure free after at least 1 year of follow-up but also undergoing an FDG-PET, and (2) a group of patients with bilateral MTS of known origin, provided that it was not autoimmune. The last group included all the patients controlled in Hospital de Bellvitge with bilateral MTS excluding patients with an autoimmune origin. Seizure classification was made according to the ILAE 2017 operational classification of seizure types (9).

Comorbid pathologies were divided into three categories: autoimmune, neurological, and psychiatric. Comorbid autoimmune diseases considered were the group of 12 autoimmune diseases that appear most frequently in epilepsy according to the population study of Ong et al. (10). The comorbid psychiatric diseases considered were mood and anxiety disorders, attention deficit hyperactivity disorder, and psychosis according to Kanner (11). Neurological comorbid pathologies considered were the rest of the neurological pathologies, apart from memory disturbances, that are produced by the same etiology that also produced the epilepsy.

The study was approved by the Ethical Committee of Bellvitge University Hospital. Informed consent was obtained from all patients.

## Material

### MRI

MRI scans were acquired using a Philips 1.5 or 3 Tesla MRI scanner (Intera, Philips Medical Systems, Amsterdam, The Netherlands) according to a standard epilepsy protocol (12). Follow-up MRI studies were done with a 3 Tesla scanner while most of the first studies had been done with a 1.5 Tesla scanner.

### Fluodeoxiglucose (FDG) PET Studies

Following FDG injection and uptake under euglycemic (overnight fasting) and standardized resting conditions (eyes open, reduced ambient noise), FDG-PET scans were acquired on a Gemini TF 64 PET/CT scanner (Philips, The Netherlands; *n* = 9; 10 min 3D acquisition starting 50 min p.i. of 280 ± 62 MBq FDG). Visual readings were performed after automatic anterior-posterior commissure line realignment on transaxial, coronal, and sagittal slices spanning the entire brain.

### FDG-PET/MRI Studies

After PET and MRI were carried out, images from the two techniques were normalized so they could be superimposed on each other with anatomical reliability, and were then co-recorded.

Cerebral MRI and FDG-PET scans were analyzed by a neurologist (JS or MF) independently of the neuroradiologist (SC), and by a nuclear medicine specialist (LR). The neuroradiologist and the nuclear medicine specialist were blinded to clinical characteristics and autoantibody status. The results from the two groups were compared and in case of discrepancies in a particular study, this was analyzed together and a consensus was obtained.

### GAD Antibodies Determination

GAD ab was analyzed in serum and CSF (when available) with enzyme-linked immunosorbent assay (ELISA) at the Hospital de Bellvitge. All patients with focal epilepsy of unknown origin are tested for GAD ab in blood serum (20–30 patients per year). Patients with acute onset are tested for GAD also in CSF. In order to confirm the results in patients with positive GAD ab, immunohistochemistry and radioimmunoassay (RIA) were performed at Hospital Clinic of Barcelona, as described elsewhere (13). Additional immunological studies were performed, including determination in serum and CSF of onco-neuronal antibodies (Hu, Yo, Ma, Tr, amphiphysin) and antibodies against neuronal surface antigens (NSA-abs). These studies were carried out in the Neuroimmunology Unit of the Hospital Clinic of Barcelona. NSA-abs were identified by immunocyto-chemistry of rat hippocampal neuronal cultures, as described elsewhere (14).

Serum titers of GAD ab were defined as high when in excess of 2,000 IU/ml. High titers are required to consider the antibody as possibly pathogenic in the neurological symptoms (15, 16). In pharmacoresistant patients several determinations were made, in some cases before and after different immunotherapies (follow-up determinations were made at the Hospital de Bellvitge using ELISA) and results were given as exact number if titers were below 2,000 UI or >2,000 UI without specifying the exact value.

### Neuropsychological Examination

A comprehensive neuropsychological examination was performed, including intelligence and memory tests. We included certain subtests (Logical Memory and Visual Reproduction) of the Wechsler Memory Scales (WMS, WMS-R, and WMS-III). Premorbid intelligence was tested with the Vocabulary subtest of the Wechsler Adult Intelligence Scales (WAIS and WAIS-III). For statistical analysis of neuropsychological variables, normalized “z” scores were computed using mean and standard deviations of the raw scores of the general population. Verbal and visual memory scores were compared with vocabulary scores individually. We consider the subjects as memory-impaired if more than one subtest “z” score was one standard deviation (SD) below the general level of intelligence (vocabulary), as proposed by Lezak (17). Memory impairment was divided into mild, moderate, or severe. Severe memory deficit was considered when subject score was below 2.5 SD and moderate between 1.5 and 2.5 SD.

### Statistical Analysis

All statistical analysis was performed using SPSS for Windows (version 22.0, SPSS Inc., Chicago, IL, U.S.A.). Categorical variables were analyzed using a one-tailed chi-square analysis (with Yates correction when warranted), and continuous data were analyzed using *t*-test or Mann-Whitney U-test, ANOVA, and Kruskal-Wallis test for non-parametrical analysis. All tests were two-tailed; *P*-values < 0.05 were considered significant.

## Results

We identified 22 patients with epilepsy and GAD ab in the Epilepsy Unit database of the Hospital Universitari de Bellvitge. From this group we selected patients with high serum titers of GAD ab (>2,000 UI/ml) and a follow-up of more than 5 years. The excluded patients were 1 patient with generalized epilepsy, 2 who died during follow-up, 1 with *status epilepticus*, and 1 with pneumonia. Another patient was lost to follow-up. Finally, 13 patients with epilepsy and high titers of GAD ab were included. Among these only 2/13 (15%) patients referred an acute debut, one with limbic encephalitis that occurred during pregnancy (together with eclampsia and Hellp syndrome) and the other with an occipital *status epilepticus* in the context of celiac disease. The rest of the patients debuted with focal epilepsy. GAD ab in CSF was analyzed in 6/13 (46%) patients and was positive in all. Intrathecal synthesis was observed in 5/6 (83%). During the follow-up, several serum GAD determinations were obtained in the patients with pharmacoresistant epilepsy, and at least two determinations were obtained in all patients. The results were always above 2,000 UI/ml. No other onco-neuronal antibodies or antibodies against neuronal surface antigens (NSA-abs) were identified.

Control patients were selected from the same database. Eight patients had bilateral mesial temporal lobe sclerosis (BMTS): 2 due to meningitis, 1 to measles encephalitis, 3 to connatal anoxia, 1 encephalitis from human immunodeficiency virus, and 1 encephalitis due to parvovirus B19. Nine patients had unilateral MTS: 7 had suffered febrile seizures and 2 did not report initial precipitating injury. As mentioned above, all UMTS were seizure-free after a temporal lobectomy. In all the control patients an immunological battery including GAD ab was performed and was negative in all cases.

Demographic and clinical characteristics of patients and controls are presented in Table 1.

**Table 1:** Patients and controls.

	**PATIENTS**	**CONTROLS**		***p***
	FE+ GAD ab 13	BMTS 8	UMTS 9	
	**YEARS (RANGE)**	**YEARS (RANGE)**	**YEARS (RANGE)**	
Age	51 (24–83)	52(39–66)	46(29–68)	NS
Age at epilepsy onset	36(11–72)	16.(2–45)	14(1–34)	0.004
Disease duration	14 (5–53)	35(18–55)	32 (8–49)	0.005
	**NUMBER (%)**	**NUMBER (%)**	**NUMBER (%)**	
Gender: Male	6 (46)	4 (57)	5 (55)	NS
**FOCAL AWARENESS**				
No awareness	5 (33)	6 (75)	0 (0)	0.045
Psychic	5 (33)	1 (14)	3 (33)	
Epigastric	1 (8)	0 (0)	4 (44)	
Taste/olfactory	0 (0)	1 (14)	0 (0)	
Somatosensorial	1 (8)	0 (0)	0 (0)	
Visual	2 (16)	0 (0)	0 (0)	
Autonomic	0 (0)	0 (0)	1 (11)	
Motor	1 (8)	0 (0)	0 (0)	
Musicogenic reflex seizures	2 (15)	0 (0)	0 (0)	NS
Seizure type (main)				0.025
FIAS	8 (61)	8 (100)	9 (100)	
FBTCS	5 (41)	0 (0)	0 (0)	
Prior history of syncopes	2 (16)	0 (0)	0 (0)	NS
Acute onset or acute relapses^*^	3 (23)	5 (62)	0 (0)	NS
Seizure-free (>1 year)	6 (46)	0 (0)	0 (0)^**^	0.003
Memory impairment	6 (46)	8 (100)	9 (100)	NS
Bilateral	1 (10)	8 (100)	2 (22)	
Dominant	4 (30)	0 (0)	3(33)	
Non-dominant	0 (0)	0 (0)	4 (44)	
Autoimmune comorbidities^***^	11 (85)	0 (0)	0 (0)	0.001
Psychiatric comorbidities	0 (0)	4 (50)	1 (11)	NS
Interictal psychosis	0 (0)	2 (24)	0 (0)	
Anxiety disorders	0 (0)	1 (12)	1 (1)	
Mood disorders	0 (0)	1 (12)	0 (0)	
Other neurological comorbidities	1 (8)	2 (12)	0 (0)	NS
Mental retardation	0 (0)	1 (12)	0 (0)	
Nistagmus	1 (8)	0 (0)	0 (0)	
Mitochondrial SFN	0 (0)	0 (0)	0 (0)	
Tumor ADK colorectal/prostatic or hepatic	3(25)	1 (12)	0 (0)	NS
**EEG (SLOW ACTIVITY)**				
TIRDA	4 (30)	8 (100)	9 (100)	0.001
FIRDA	3 (23)	0 (0)	0 (0)	NS
**EEG, IED**				
Unilateral	3 (23)	3 (37)	9 (100)	NS
Temporal (>80%)	1 (8)	5 (62)	9 (100)	NS
Bilateral temporal	7 (53)	4 (50)	0 (0)	NS
Extratemporal	5 (33)	1 (12)	0 (0)	0.01
Absence of IED	6 (46)	0 (0)	0 (0)	0.003

*Electroclinical and demographic characteristics. FE+GAD ab: focal epilepsy with glutamic acid decarboxylase antibodies, BMTS: bilateral mesial temporal lobe sclerosis, UMTS: unilateral mesial temporal lobe sclerosis. FIAS: focal impaired awareness seizure, FBTCS: focal to bilateral tonic-clonic seizure. LE: limbic encephalitis. Autoinmune diseases reported: DM1, Hypothyroidism, psoriasis, celiac disease and myasthenia. ADK: adenocarcinoma. SFN: small fiber neuropathy. EEG: electroencephalogram. TIRDA: temporal rhythmic delta activity. FIRDA: frontal rhythmic delta activity, IED: interictal epileptiform discharges*.

* *Considered acute onset: Status epilepticus, limbic encephalitis or viral or bacterial encephalitis or meningitis*.

** *Prior to epilepsy surgery*.

*** *One or more autoimmune diseases per patient*.

No differences were observed between patients and controls in terms of gender and age. Patients reported earlier epilepsy onset compared to controls (*p*: 0.004), both UMTS and BMTS, and with a shorter disease duration (*p*: 0.005). After a mean disease duration of 14 years (range 6–53 years), nearly half of the patients with FE +GAD ab−6/13 (46%)—were seizure-free. Interestingly only one of the patients who was seizure-free received immunosuppressive agents, due to a liver transplant. Half of the patients with drug-resistant FE+ GAD ab had received immunotherapy (corticoids, IVIG, azathioprine, cyclophosphamide, or mycophenolate) without significant improvement in seizure frequency. All control patients with UMTS were seizure-free after surgery while none of those with BMTS were. BMTS patients suffered weekly or monthly seizures.

In comparing the seizure characteristics of FE+ GAD ab patients with those of controls (BMTS or UMTS) some differences were found: somatosensorial, motor, and visual focal aware seizures were only reported by patients with FE +GAD ab (4/13: 30%, p:0.045), suggesting a symptomatogenic zone beyond the temporal lobe. In addition, some patients reported different seizure types during the disease evolution. One man had seizures with cephalic parestesias and right focal motor clonic seizures during the first months of the disease. This seizure type completely disappeared and afterwards he started to present a déjà-vu sensation preceding a focal impaired awareness seizure. Another man reported visual focal aware seizures only at disease onset while later on this seizure type completely disappeared. Considering seizure types, focal to bilateral tonic-clonic seizures (FBTCS) as the main seizure type were only reported by patients with FE + GAD ab, and they occurred predominantly during night sleep. On the other hand, focal impaired awareness seizures (FIAS) were the predominant seizure type in controls (BMTS and UMTS) (*p*: 0.025). Two patients with FE +GAD ab reported musicogenic reflex seizures in addition to non-provoked FIAS and FBTCS. None of the controls mentioned any kind of reflex seizures (*p*: NS).

Apart from seizures, a previous history of syncope was reported in 2/13 (16%) of the patients with FE +GAD ab. None of them had a history of cardiac disorders. One of the patients, a man aged 83, was diagnosed at 70 with carotid sinus hypersensitivity and a pacemaker was implanted. He suffered no more syncopes but 2 years later he started suffering FIAS. Now on antiepileptic drugs (lamotrigine 300 mgr/day), his seizures have been completely controlled although he suffers from progressive verbal memory loss.

Comparing EEG characteristics of patients and controls, a more widespread distribution on interictal epileptiform discharges (IED) was observed in FE+ GAD ab patients than in controls, predominantly in the frontopolar and frontocentral regions, but also in the occipital and parietal regions (*p*:0.01). Rhythmic delta activity was observed in all controls in anterior temporal lobes (affecting one or both) while in patients this was less frequent (*p*: 0.001) and appeared in anterior temporal lobe areas and in frontal lobe areas (in two patients only in frontal lobe areas). Six patients (46%) had no IED even in prolonged VEEG study. Ictal EEG was obtained in all controls and in three FE+GAD ab patients. In 2/3 patients it was suggestive of unilateral temporal anterior seizures while in 1/3 bilateral independent anterior temporal lobe seizures were recorded.

Memory impairment was observed in 8/13 (61%) of the patients with FE+ GAD ab; it was moderate in three patients. Interestingly, in three of the 8 patients seizures were completely controlled. Memory decline over the last 8 years was documented in 4/8 (50%) FE+GAD ab patients. All 8/8 (100%) control patients with BMTS had moderate or severe memory impairment, both verbal and visual, with other neuropsychological deficits mostly affecting frontal lobe structures. Control patients with UMTS had mild or moderate verbal or visual memory impairment, with mild or moderate verbal memory impairment in 3/9 (33%), mild or moderate visual memory impairment in 4/9 (44%), and mild or moderate bilateral memory impairment in 2/9 (22%).

Comparing comorbidities, patients with FE+GAD ab tended to suffer other autoimmune diseases (T1DM being the most frequent) (*p*: 0.001) while patients with BMTS or UMTS tended to suffer more psychiatric comorbidities although this last difference was not statistically significant.

### MRI Characteristics (see Table 2, Figures 1, 2)

In 5 (38%) FE+GAD ab patients the first available MRI was performed within the first year from the epilepsy debut. In one patient (1/5; 20%) the initial MRI, done in the first 3 months, showed bilateral amygdalar and hippocampal hyperintensities without atrophy. Greater hyperintensities were found in amygdalar tissue compared with hippocampus (patient previously reported) (3). In the rest (4/5; 80%) the first MRI was normal.

**Table 2:** Clinical and neuroimaging characteristics of FE+ GAD ab patients at debut and during follow-up.

**Patient**	**Age at epilepsy onset**	**Acute onset**	**Follow up (years)**	**Seizure semiology (focal awareness)**	**Initial MRI**	**Follow-up MRI 1**	**Follow-up MRI 2**	**Follow-up MRI 3**	**FDG-PET**	**Follow-up FDG-PET**
1	26	Yes	10	Déjà vu, fear	Hippocampal and amygdalar hyperintensities and volume increase	(3 years) Normal	(7 years) Normal	(11 years) Normal	(8 years) MRTL hypermetabolism MLTL hypometabolism	(9 years) MRTL and MLTL hypometabolism
2	38	No	8	Initial: right motor Follow-up: déjà vu, MRS	Normal		(6 years) Normal		(6 years) Left insular and MRTL hypometabolism	(8 years) Bilateral insular and MTL hypometabolism
3	41	No	13	Déjà vu	Normal	(10 year) Normal	(10 year) Normal	(13 year) L. hippocampal atrophy	(5 years) MLTL hypometabolism	
4	72	No	13	No awareness FBTCS		(1 year) Normal	Pacemaker was implanted		(10 years) Bilateral MTL and insular hypometabolism	
5	30	Yes	11	Initial: visual Follow-up: déjà vu, epigastric		(4 years) Normal	(7 years) Normal	(10 years) R. hippocampal atrophy	(7 years) Bilateral MTL and left insular hypometabolism	
6	11	No	56	Paresthesia in right arm		(46 years) Normal			(52 years) Left Rolandic and insular hypometabolism	
7	50	Yes	18	No awareness FBTCS		(11 years) Left hippocampal sclerosis	(13 years) Left hippocampal sclerosis Right hippocampal aedema, hyperintensity and contrast enhancement	(15 years) Bilateral hippocampal sclerosis	(14 years) Bilateral MTL hypermetabolism Bilateral insular hypometabolism	(15 years) Bilateral MTL hypometabolism Bilateral insular hypometabolism
8	35	No	8	No awareness FBTCS		(3 years) Normal			(3 years) Bilateral MTL hypometabolism	
9	11	No	15	Visual		(8 years) Normal	(12 years) Normal	(15 years) Normal	(12 years) Bilateral MTL hypometabolism	
10	41	No	20	No awareness FBTCS		(8 years) Normal				
11	62	No	7	No awareness FBTCS	Normal					
12	39	No	31	Epigastric MRS		(11 years) Normal	(21 years) Normal	(31 years) R. hippocampal atrophy	(27 years) RMTL hypometabolism	
13	24	No	7	Déjà vu	Normal	(2 years) L. hippocampal sclerosis	(5 years) L. hippocampal sclerosis		(5 years) LMTL hypometabolism	

*MRTL, mesial right temporal lobe; MLTL, mesial left temporal lobe; MRS, musicogenic reflex seizures; FBTCS, focal to bilateral tonic-clonic seizure; Hippocampal sclerosis, atrophy and hyperintensity in the hippocampus*.

**Figure 1:**
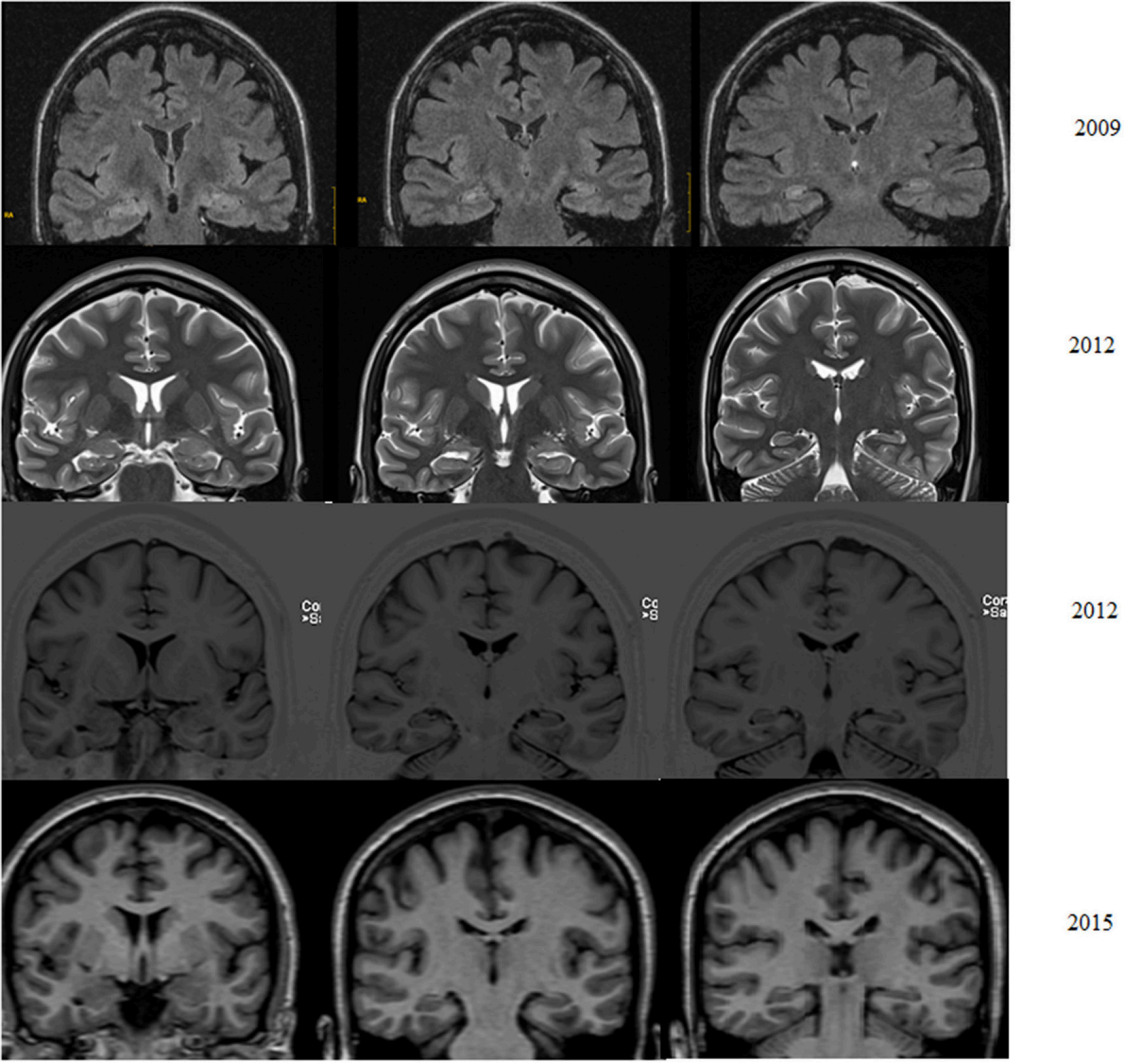
Patient with pharmacoresistant temporal lobe epilepsy from age 25. Five years later suffers weekly focal impaired awareness seizures but no memory decline. Left hippocampal tail atrophy is found on 2012 MRI and confirmed in 2015 (Coronal T1).

**Figure 2:**
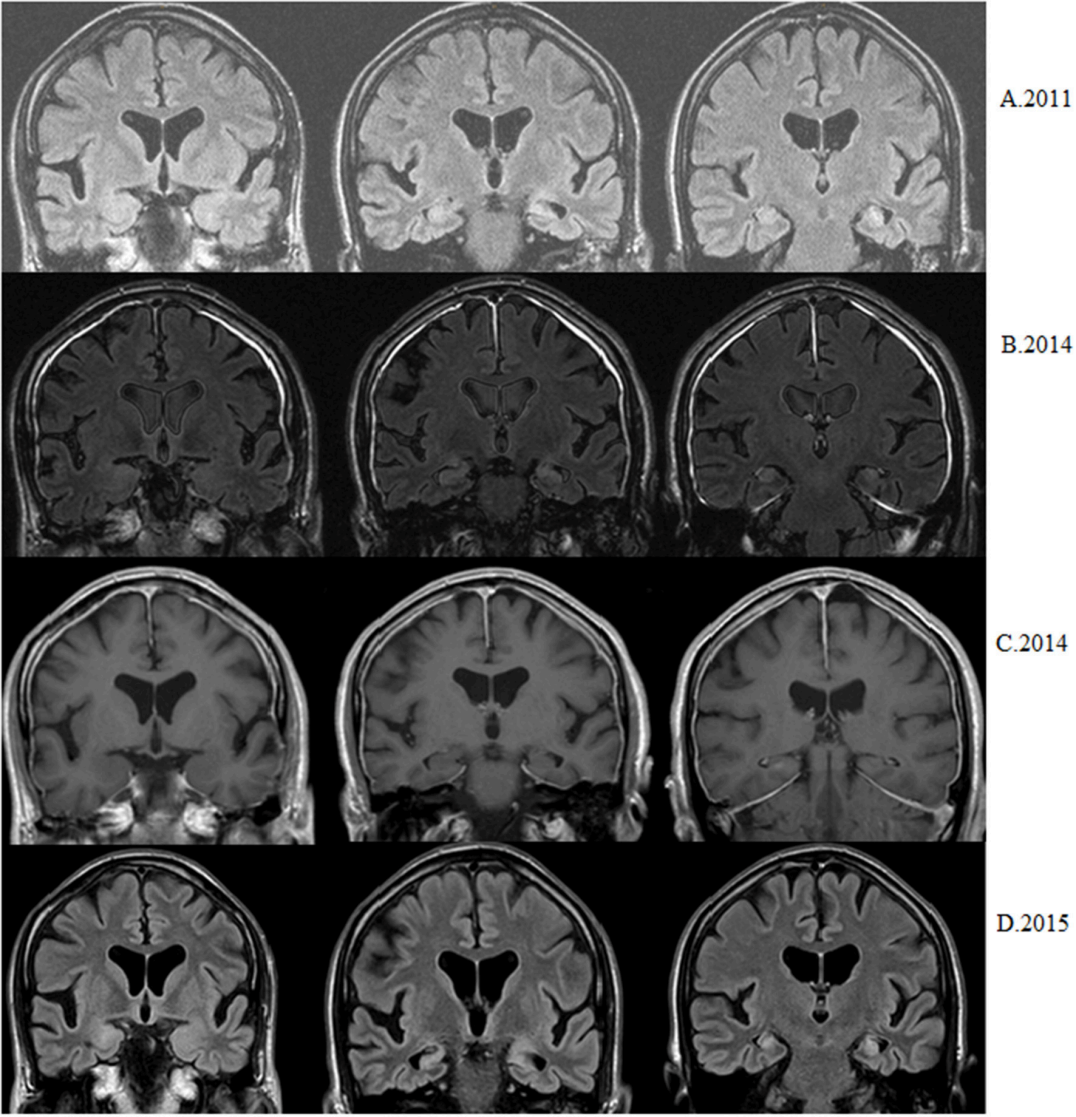
Patient with epilepsy debut at 56. Nowadays seizure-free but with progressive memory decline. **(A)** First available MRI shows left hippocampal atrophy and hyperintensity (coronal flair). **(B)** Three years later bilateral hippocampal hyperintensities are observed without right hippocampal atrophy (coronal flair). **(C)** Three years later nodular right hippocampal contrast enhancement is observed (coronal T1 after injection of gadolinium). **(D)** After 6 months bilateral hippocampal sclerosis (atrophy+ hyperintensity) is observed (coronal flair).

A follow-up MRI was performed in all patients. In 8 (61%) FE+GAD ab patients several (>3) MRI studies were done. The patient with bilateral amygdalar-hippocampal hyperintensities but no atrophy, when scanned 11 years later, had a normal MRI without hippocampal atrophy. Subtle unilateral hippocampal sclerosis (predominantly affecting the hippocampal tail) was found in 4 (80%) patients with an initially normal MRI. Three of them had a disease duration of between 5 and 10 years and the other a disease duration >30 years.

In a patient whose first available MRI was done 11 years after disease onset, left hippocampal sclerosis was observed. Thirteen years later the patient suffered an acute episode of limbic encephalitis (probably a relapse) affecting the right hippocampus and evolving to a bilateral MTS within a year.

In only three controls was an initial MRI available (carried out within the first year from epilepsy debut). In two, both suffering encephalitis, bilateral hippocampal/amygdalar hyperintensities were found, while in the other initial MRI was normal. In all, after <3 months bilateral hippocampal atrophy was observed.

### FDG- PET Characteristics (see Tables 2, 3 and Figure 3)

Initial FDG-PET was available in 2 patients because both experienced an acute disease onset or had a relapse. In both cases unilateral temporal lobe hypermetabolism was observed. In one patient the hypermetabolism persisted for 6 months despite immunosuppression therapy (corticoids, IVIG, and monthly cyclophosphamide).

**Table 3:** Correlation between MRI findings and FDG-PET findings in patients with focal epilepsy + GAD ab.

** 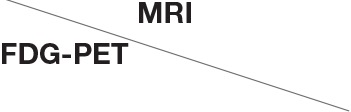 **	**Normal**	**Unilateral HIP atrophy**	**Bilateral HIP atrophy**
Unilateral hypometabolism		3 (27%)	
Unilateral insular hypometabolism	1 (9%)		
Bilateral HIP hypometabolism	2 (18%)	1 (9%)	
Bilateral HIP + unilateral insular hypometabolism		1 (9%)	
Bilateral HIP+bilateral insular hypometabolism	2 (18%)		1 (9%)

*HIP, Hippocampal, MRI, magnetic resonance imaging, FDG-PET, fluoxideoxiglucose positron emission tomography*.

**Figure 3:**
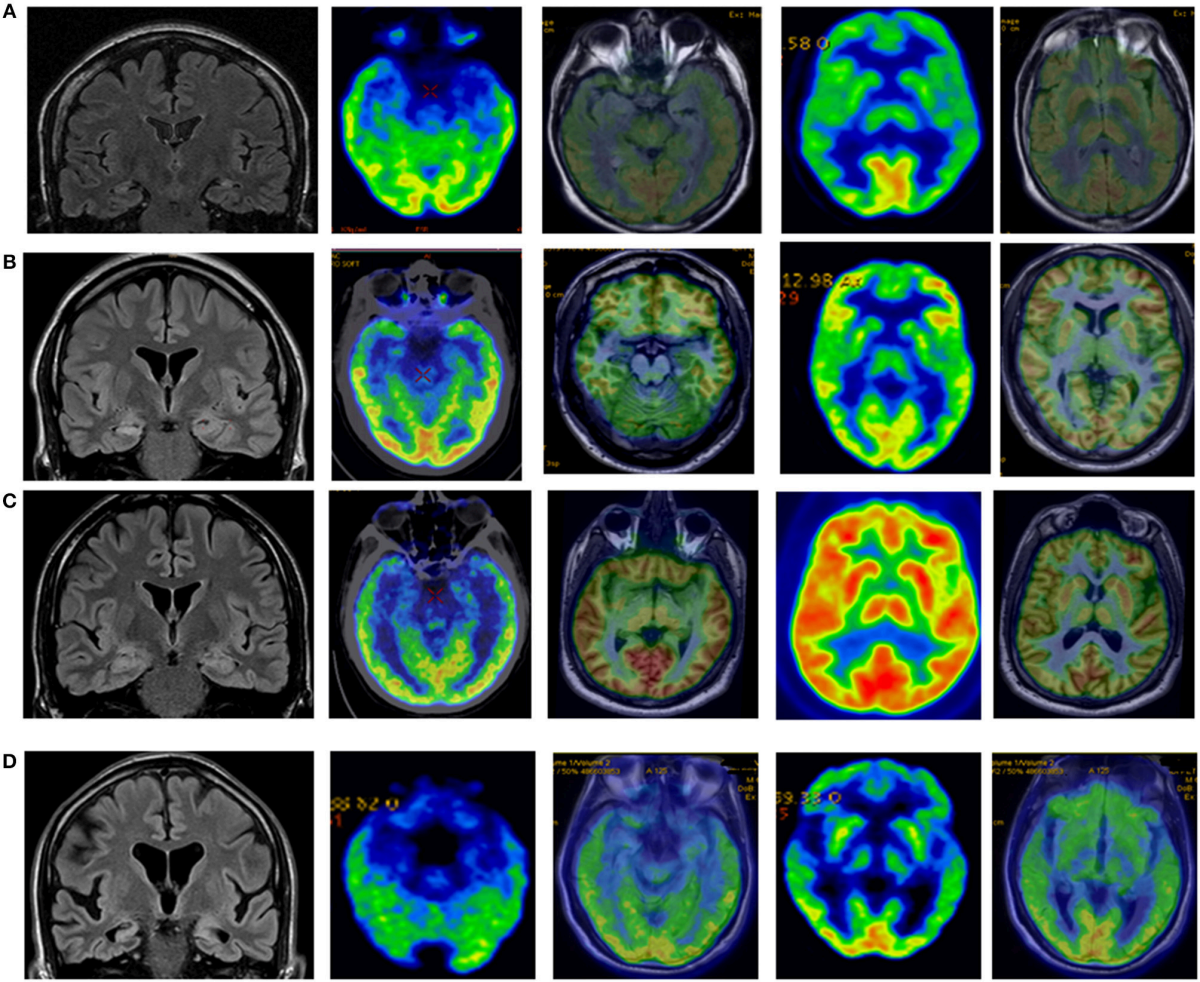
**(A)** Control patient who suffered measles encephalitis at 2. Epilepsy started at 8. Currently (more than 20 years later) suffers weekly seizures and severe memory deficits. MRI shows bilateral hippocampal atrophy and FDG PET bilateral medial temporal lobe hypometabolism with normal insular metabolism. **(B)** Control patient who suffered febrile seizures. Epilepsy started at 9. He suffered weekly FIAS preceded by non-specific sensation. MRI right hippocampal hyperintensity and FDG-PET right mesial hypometabolism with normal insular metabolism. **(C)** Patient with T1DM from age 29 and temporal lobe epilepsy from age 30. MRI remains normal after 5 years but FDG-PET shows bilateral medial temporal lobe hypometabolism and left insula hypometabolism. **(D)** Patient with epilepsy from age 56. Seizure-free but progressive and severe memory decline. MRI shows bilateral hippocampal sclerosis and insular atrophy, and FDG-PET shows bitemporal medial hypometabolism and bilateral insular hypometabolism.

A follow up FDG-PET was done in 11/13 (84.6%) patients more than 5 years after epilepsy debut. In 2 patients more than one FDG-PET studies was performed.

All patients had an abnormal FDG-PET study. Mesial temporal lobe hypometabolism was observed in 10/11 (91%) patients; it was bilateral in 8 (72%) and unilateral in 3 (27%). Moreover, insular hypometabolism was observed in 5/11 (45%) patients; it was bilateral in 3 (27%). In all patients except one, insular hypometabolism was observed together with mesial temporal lobe hypometabolism. Isolated unilateral insular hypometabolism was observed in one patient—a man with somatosensorial focal awareness and normal MRI.

### Comparing FDG-PET Findings to MRI Findings in Patients (see Table 3)

At the end of follow-up 5 patients had a normal MRI (without hippocampal atrophy) but none of them had a normal FDG-PET. Among 5 FE+GAD ab patients who went on to show unilateral hippocampal atrophy, 3 (60%) had ipsilateral hippocampal hypometabolism and 2 (40%) bilateral hippocampal hypometabolism, with associated insular hypometabolism in one. Interestingly, 2 FE+GAD ab patients (27%) with a normal MRI showed bilateral hippocampal hypometabolism, which was associated with insular hypometabolism in one. The patients with bilateral hippocampal sclerosis also had bilateral hippocampal hypometabolism and bilateral insular hypometabolism. Overall, only 3 patients (27%) had strictly unilateral mesial temporal involvement (considering FDG-PET and MRI findings).

### Comparing FDG- PET Findings of FE + GAD ab Patients and Controls (see Figure 3)

FDG-PET was performed in 7/8 (88%) BMTS controls and in 9/9 (100%) UMTS controls. Bilateral mesial temporal lobe hypometabolism was observed in all BMTS controls, and unilateral or bilateral mesial temporal lobe hypometabolism in all UMTS patients, whereas insular hypometabolism was not observed in any (*p*: 0.002).

## Discussion

In our study both clinical and paraclinical findings suggest that FE +GAD ab involves limbic areas but also the insular region, and in one patient this lobe was the only one involved.

Clinical symptoms suggestive of insular epilepsy include occurrence in full consciousness of a symptomatic sequence associating laryngeal discomfort with thoracic oppression or dyspnoea, unpleasant paraesthesia—a warm sensation focused on the perioral region or extending to a large somatic territory, and dysarthria, or dysphonic, speech defects (18). One of the patients reported seizures as an unpleasant paresthesia starting in his left arm and rapidly affecting face and tongue, with the left half of his body always in full awareness. A very specific form of sensitive focal aware seizures (pilomotor seizures) have been reported in patients with limbic encephalitis. These are probably also suggestive of insular involvement (19). Two recent case series have also suggested that insular semiology (especially other autonomic semiology) is a part of pilomotor seizures, while perisylvian semiology is highly suggestive of autoimmune epilepsy related to GAD ab or to other antibodies (20–22). Bradyarrhythmia has recently been described as a distinctive prodrome of voltage-gated potassium channel complex/leucine-rich glioma inactivated 1 antibody encephalitis (VGKC/LGI1–ab) leading to pacemaker implantation in 3 cases (23), just as in one of our patients.

The insular region is considered to be one of the cortical autonomic areas (24). GABAergic neurons have been found in several brain regions involved in the control of cardiorespiratory function (25) including the nucleus tractus solitarius (NTS) (26) and the ventrolateral medulla (27). GABA is known to play a vital role in several pacemaker networks. The most extensively studied pacemaker network is in the suprachiasmatic nucleus of the hypothalamus, an area that is responsible for controlling food intake and sleep as well as regulating body temperature and heart rate (28). We hypothesize that a loss of GABAergic neurons due to GAD ab, especially in certain autonomic brain areas (hypothalamus, insula), could affect the modulation of heart rate.

Functional neuroimaging studies also support the hypothesis of an insular involvement in patients with FE +GAD ab. In most of our patients FDG-PET showed hypometabolism in MTLE structures, but also insular hypometabolism in nearly 40% of patients. No insular hypometabolism was found in controls. Another case report showed abnormal extratemporal glucose metabolism (in this case hypermetabolism) in a patient with FE+ GAD ab (29). FDG-PET scans have been demonstrated to reliably lateralize seizure focus in MTLE, with decreased glucose uptake in the epileptogenic temporal lobe (30). It is generally believed that the region of hypometabolism is larger than the epileptogenic zone, and some studies (31) have suggested that insular hypometabolism, most frequently ipsilateral to the hippocampal sclerosis, is due to seizure propagation and does not influence seizure outcome after surgery. Other studies (32–34) have found a relationship between extratemporal hypometabolism in MTLE patients (mainly insula and frontal lobe) with poorer surgical outcome. Case studies are nowadays suggesting that pharmacoresistant FE+ GAD ab has a poor surgical outcome (35, 36). Bitemporal involvement and extratemporal involvement (insular) could be an explanation for this poor surgical outcome. In addition, insular involvement could be an important diagnostic clue in patients with MTLE epilepsy of unknown origin, by raising suspicion of an autoimmune origin due to GAD ab.

In our group of patients with FE + GAD ab, two reported suffering musicogenic reflex seizures (MRS) [Patients previously published (37)]. MRS may be a distinctive seizure type in patients with epilepsy and GAD ab.

Little information is available about long-term follow-up of patients with FE + GAD ab. In a recent study no hippocampal sclerosis (HS) was observed during the follow-up in 12 patients; however, only one of them was followed up for more than 5 years (35). In the study of Wagner et al. (6) no hippocampal volume loss was observed after two years. In another recent study which included 19 patients with FE+ GAD ab, hippocampal abnormalities were found in <30% of patients (38). In our study, where all patients were followed up for over 5 years and in the case of 2 patients more than 30 years, slight unilateral hippocampal atrophy was observed in nearly half of them and bilateral hippocampal atrophy in one. The most important finding in the study of Fredriksen et al. (38) was that patients had a disproportionate parenchymal atrophy with age, again suggesting a widespread disease not limited to the temporal lobe.

Interestingly, an anatomopathological study including 2 patients who underwent temporal lobectomy for medically refractory epilepsy + GAD ab (9 and 10 years follow-up) observed an ILAE type 3 HS or CA4 predominant HS, without lymphocytes or plasma cell infiltration (39). ILAE type 3 HS predominantly affects CA4 and the dentate gyrus. ILAE type 3 HS is the least common form of HS; patients with this form of HS typically develop epilepsy at a later age, often in the absence of an initial precipitating injury or identifiable etiology (40).

Three of our patients reported progressive memory decline even when their epilepsy was well-controlled. Tagaki et al. (41) conducted a case control study comparing cognitive performance of late-onset type one diabetic (LADA) patients with GAD ab and matched type 2 diabetes mellitus patients. They observed that verbal and visual test scores were lower in LADA+GAD ab patients and hypothesized that GAD-positive diabetic patients had an increased risk of cognitive decline compared to patients with type 2 diabetes of comparable diabetic severity.

In addition, insular atrophy and an abnormal insula functional network are also related to memory loss found in early stages of Alzheimer disease and can be a marker of progression to Alzheimer disease in patients with mild amnestic cognitive impairment (42). Both progressive insular and hippocampal damage might account for the memory decline observed in our patients.

## Conclusions

Epilepsy with GAD ab affects the limbic system unilaterally and, more frequently, bilaterally. Clinical, EEG, and FDG-PET findings suggest a widespread disease not restricted to the temporal lobe.

Progressive memory decline together with progressive hippocampal damage may be observed even in patients with well-controlled epilepsy.

Insular hypometabolism is only observed in epilepsy patients with GAD ab and not in controls with unilateral or bilateral MTS, so it may be an important diagnostic clue.

## Study limitations

Our study has some limitations, notably the low number of patients included and the selection bias in forming the two control groups. We included all patients with BMTS followed up in our center with sufficient information, and also those with the “purest” UMTS. However, the most important limitations are probably the lack of initial MRI studies in some patients and most of the controls, and the lack of follow-up studies in patients with well-controlled epilepsy. Moreover, but no less important, the use of different MRI scanners (the first studies were done with a 1.5T scanner and the following studies with a 3T scanner) could have led to an underestimation of mild hippocampal atrophies in the first studies.

Larger studies with longer follow-up are needed to confirm our initial findings.

## Author Contributions

MF and MC conceived and designed the study. MF, SJ, CC, JM, and JB analyzed the data. SC, JS-P, LR-B, and MF analyzed MRI, FDG-PET, and MRI/PET images. MF and FM analyzed GAD determinations. MF and MC wrote the paper.

### Conflict of Interest Statement

The authors declare that the research was conducted in the absence of any commercial or financial relationships that could be construed as a potential conflict of interest.

## References

[1] Markram H Toledo-Rodriguez M Wang Y Gupta A Silberberg G Wu C. Interneurons of the neocortical inhibitory system. Nat Rev Neurosci. (2004) 5:793–80. 10.1038/nrn1519 15378039

[2] Saiz A Blanco Y Sabater L González F Bataller L Casamitjana R. Spectrum of neurological syndromes associated with glutamic acid decarboxylase antibodies: diagnostic clues for this association. Brain (2008) 131:2553–63. 10.1093/brain/awn183 18687732

[3] Falip M Miro J Carreño M. Saiz A Villanueva V Quilez A Prevalence and immunological spectrum of temporal lobe epilepsy with glutamic acid decarboxylase antibodies. Eur J Neurol. (2012) 19:827–33. 10.1111/j.1468-1331.2011.03609.x 22353320

[4] Monnerat BZ Rodrigues T Nakano FN Júnior AV Martins AP Sakamoto AC. Opercular myoclonic-anarthric status epilepticus due to antibody-associated encephalitis. Epileptic Disord. (2013) 15:342–6. 10.1684/epd.2013.0596 23981587

[5] Martin RC Sawrie SM Knowlton RC Bilir E Gilliam FG Faught E. Bilateral hippocampal atrophy: consequences to verbal memory following temporal lobectomy. Neurology (2001) 57:597–604. 10.1212/WNL.57.4.597 11524466

[6] Wagner J Schoene-Bake JC Malter MP Urbach H Huppertz HJ Elger CE. Quantitative FLAIR analysis indicates predominant affection of the amygdala in antibody-associated limbic encephalitis. Epilepsia (2013) 54:1679–87. 10.1111/epi.12320 23889589

[7] Wagner J Weber B Elger C. Helmstaedter C Malter MP Stoecker W Early and chronic grey matter and volume changes in limbic encephalitis revealed by voxel-based morphometry. Epilepsia (2015) 56:754–61. 10.1111/epi.13297 25809952

[8] Fauser S Uttner I Ariño H Scherbaum A Saiz A Lewerenz J. Long latency between GAD-antibody detection and development of limbic encephalitis – a case report. BMC Neurol. (2015) 15:177–81. 10.1186/s12883-015-0435-9 26420440 PMC4589124

[9] Fisher R. Cross H. D'Souza C. Higurashi N Hirsch E Jansen FE Instruction manual for the ILAE 2017 operational classification of seizure types. Epilepsia (2017) 58:531–42. 10.1111/epi.13670 28276064

[10] Ong MS Kohane IS Cai T Gorman MP Mandl KD. Population-level evidence for an autoimmune etiology of epilepsy. JAMA Neurol. (2014) 71:569–74. 10.1001/jamaneurol.2014.188 24687183 PMC4324719

[11] Kanner A Psychiatric comorbidities and epilepsy: is it the old story of the chicken and the egg? Ann Neurol. (2012) 72:153–5. 10.1002/ana.23679 22926848

[12] Wellmer J Quesada CM Rothe L Elger CE Bien CG Urbach H. Proposal for a magnetic resonance imaging protocol for the detection of epileptogenic lesions at early outpatient stages. Epilepsia (2013) 54:1977–87. 10.1111/epi.12375 24117218

[13] Saiz A Arpa J Sagasta A Casamitjana R Zarranz JJ Tolosa E. Autoantibodies to glutamic acid decarboxylase in three patients with cerebellar ataxia, late-onset insulin-dependent diabetes mellitus, and polyendocrine autoimmunity. Neurology (1997) 49:1026–30. 10.1212/WNL.49.4.1026 9339684

[14] Ances BM Vitaliani R Taylor RA Liebeskind DS Voloschin A Houghton DJ. Treatment responsive limbic encephalitis identified by neuropil antibodies: MRI and PET correlates. Brain (2005) 128:1764–77. 10.1093/brain/awh526 15888538 PMC1939694

[15] Peltola J Kulmala P Isojärvi J Saiz K Latvala J Palmio K. Autoantibodies to glutamic acid decarboxylase in patients with therapy-resistant epilepsy. Neurology (2000) 55:46–50. 10.1212/WNL.55.1.46 10891904

[16] Giometto B Nicolao P Macucci M Tavolato B Foxon R Bottazzo GF. Temporal-lobe epilepsy associated with glutamic-acid-decarboxylase autoantibodies. Lancet (1998) 352:457. 10.1016/S0140-6736(05)79192-3 9708763

[17] Lezak MD Gray DK. Sampling problems and nonparametric solutions in clinical neuropsychological research. J Clin Neuropsychol. (1984) 6:101–6. 10.1080/01688638408401200 6699182

[18] Isnard J Guénot M Sindou M Mauguiere F. Clinical manifestations of insular lobe seizures: a stereo-electroencephalographic study. Epilepsia (2004) 45:1079–90. 10.1111/j.0013-9580.2004.68903.x 15329073

[19] Rocamora R Becerra JL Fossas P Gomez M Vivanco-Hidalgo RM Mauri JA Pilomotor seizures: an autonomic semiology of limbic encephalitis? Seizure (2014) 23:670–3. 10.1016/j.seizure.2014.04.013 24890932

[20] Gillinder L Tjoa L Mantzioris B Blum S Dionisio S. Refractory chronic epilepsy associated with neuronal auto-antibodies: could perisylvian semiology be a clue? Epileptic Disord. (2017) 19:439–49. 10.1684/epd.2017.0946 29258968

[21] Baysal-Kirac L Tuzun E Erdag E Ulusoy C Vanli-Yavuz EN Ekizoglu E. Neuronal autoantibodies in epilepsy patients with peri-ictal autonomic findings. J Neurol. (2016) 263:455–66. 10.1007/s00415-015-8002-2 26725084

[22] Najjar S Pearlman D Najjar A Ghiasian V Zagzag D Devinsky O. Extralimbic autoimmune encephalitis associated with glutamic acid decarboxylase antibodies: an underdiagnosed entity? Epilepsy Behav. (2011) 21:306–13. 10.1016/j.yebeh.2011.03.038 21620774

[23] Naasan G Irani S Bettcher B Geschwind M Gelfand J. Episodic bradycardia as Neurocardiac prodrome to voltage-gated potassium channel complex/leucine-rich glioma inactivated-1 antibody encephalitis. JAMA Neurol. (2014) 71:1300–4. 10.1001/jamaneurol.2014.1234 25133690 PMC4474144

[24] Samuels MA. The brain-heart connection. Circulation (2007) 116:77–84. 10.1161/CIRCULATIONAHA.106.678995 17606855

[25] Dehkordi O Millis RM Dennis GC Jazini E Williams C Hussain D. Expression of alpha-7 and alpha-4 nicotinic acetylcholine receptors by GABAergic neurons of rostral ventral medulla and caudal pons. Brain Res. (2007) 1185:95–102. 10.1016/j.brainres.2007.09.027 17950703

[26] Wang J Irnaten M Mendelowitz D. Characteristics of spontaneous and evoked GABAergic synaptic currents in cardiac vagal neurons in rats. Brain Res. (2001) 889:78–83. 10.1016/S0006-8993(00)03112-7 11166689

[27] Favero MT Takakura AC de Paula PM Colombari E Menani JV Moreira TS. Chemosensory control by commissural nucleus of the solitary tract in rats. Respir Physiol Neurobiol. (2011) 179:227–34. 10.1016/j.resp.2011.08.010 21884826

[28] Aton S Huettner J Straume M Herzog ED. GABA and Gi/o differentially control circadian rhythms and synchrony in clock neurons. Proc Natl Acad Sci USA. (2006) 103:19188–93. 10.1073/pnas.0607466103 17138670 PMC1748197

[29] Kojima G Inaba M Bruno MK. PET-positive extralimbic presentation of anti-glutamic acid decarboxylase antibody-associated encephalitis. Epileptic Disord. (2014) 16:358–61. 10.1684/epd.2014.0666 25042574

[30] Theodore WH Newmark ME Sato S Brooks R Patronas N De La Paz R. 18F. Fluorodeoxyglucose positron emission tomography in refractory complex partial seizures. Ann Neurol. (1983) 14:429–37. 10.1002/ana.410140406 6416141

[31] Bouilleret V Duphont S Spelle L Baulac M Samson Y Semah F. Insular cortex involvement in mesiotemporal lobe epilepsy: a positron emission tomography study. Ann Neurol. (2002) 51:202–8. 10.1002/ana.10087 11835376

[32] Wong CH Bleasel A Wen L Eberl S Byth K Fulham M. The topography and significance of extratemporal hypometabolism in refractory mesial temporal lobe epilepsy examined by FDG-PET. Epilepsia (2010) 51:1365–73. 10.1111/j.1528-1167.2010.02552.x 20384730

[33] Vinton A Carne R Hicks R Desmond PM Kilpatrick C Kaye AH. The extent of resection of FDG-PET hypometabolism relates to outcome of temporal lobectomy. Brain (2007) 130:548–60. 10.1093/brain/awl232 16959818

[34] Koutroumanidis M Hennessy MJ Seed PT Elwes RD Jarosz J Morris RG. Significance of interictal bilateral temporal hypometabolism in temporal lobe epilepsy. Neurology (2000) 54:1811–21. 10.1212/WNL.54.9.1811 10802790

[35] Malter MP Frisch C Zeitler H Surges R Urbach H Helmstaedter C. Treatment of immune-mediated temporal lobe epilepsy with GAD antibodies. Seizure (2015) 30:57–63. 10.1016/j.seizure.2015.05.017 26216686

[36] Carreño M Bien CG Asadi-Pooya AA Sperling M Marusic P Elisak M. Epilepsy surgery in drug resistant temporal lobe epilepsy associated with neuronal antibodies. Epilepsy Res. (2017) 129:101–5. 10.1016/j.eplepsyres.2016.12.010 28043058 PMC5285301

[37] Falip M Rodriguez-Bel L Castañer S Miro J Jaraba S Mora J. Musicogenic reflex seizures in epilepsy with glutamic acid decarbocylase antibodies. Acta Neurol Scan (2018) 137:272–6. 10.1111/ane.12799 28766694

[38] Fredriksen JR Carr CM Koeller KK Verdoorn JT Gadoth A Pittock SJ Kotsenas AL. MRI findings in glutamic acid decarboxylase associated autoimmune epilepsy. Neuroradiology (2018) 60:239–45. 10.1007/s00234-018-1976-6 29353399

[39] Glover R DeNiro LV Lasala PA Weidenheim KM Graber JJ Boro A. ILAE type 3 hippocampal sclerosis in patients with anti-GAD-related epilepsy. Neurol Neuroimmunol Neuroinflamm. (2015) 2:e122. 10.1212/NXI.0000000000000122 26161431 PMC4484895

[40] Blümcke I Thom M Aronica E Armstrong DD Bartolomei F Bernasconi A. International consensus classification of hippocampal sclerosis in temporal lobe epilepsy: a Task Force report from the ILAE Commission on Diagnostic Methods. Epilepsia (2013) 54:1315–29. 10.1111/epi.12220 23692496

[41] Takagi M Ishigaki Y Uno K Sawada S Imai J Kaneko K. Cognitive dysfunction associated with anti-glutamic acid decarboxylase autoimmunity: a case-control study. BMC Neurol. (2013) 13:76. 10.1186/1471-2377-13-76 23835051 PMC3711917

[42] Xie CH Bai F Yu H Shi Y Yuan Y Chen G. Abnormal insula functional network is associated with episodic memory decline in amnestic mild cognitive impairment. Neuroimage (2012) 63:320–7. 10.1016/j.neuroimage.2012.06.062 22776459 PMC4513936

